# A three-stage, deep learning, ensemble approach for prognosis in patients with Parkinson’s disease

**DOI:** 10.1186/s13550-021-00795-6

**Published:** 2021-06-07

**Authors:** Kevin H. Leung, Steven P. Rowe, Martin G. Pomper, Yong Du

**Affiliations:** 1 Department of Biomedical Engineering, Johns Hopkins University School of Medicine, 601 N Caroline St. JHOC 4263, Baltimore, MD 21287 USA; 2 The Russell H. Morgan, Department of Radiology and Radiological Science, Johns Hopkins University School of Medicine, Baltimore, MD 21287 USA

**Keywords:** Parkinson’s disease, Deep learning, Ensemble learning, DaTscan, Prognosis

## Abstract

**Background:**

Diagnosis of Parkinson’s disease (PD) is informed by the presence of progressive motor and non-motor symptoms and by imaging dopamine transporter with [^123^I]ioflupane (DaTscan). Deep learning and ensemble methods have recently shown promise in medical image analysis. Therefore, this study aimed to develop a three-stage, deep learning, ensemble approach for prognosis in patients with PD.

**Methods:**

Retrospective data of 198 patients with PD were retrieved from the Parkinson’s Progression Markers Initiative database and randomly partitioned into the training, validation, and test sets with 118, 40, and 40 patients, respectively. The first and second stages of the approach extracted features from DaTscan and clinical measures of motor symptoms, respectively. The third stage trained an ensemble of deep neural networks on different subsets of the extracted features to predict patient outcome 4 years after initial baseline screening. The approach was evaluated by assessing mean absolute percentage error (MAPE), mean absolute error (MAE), Pearson’s correlation coefficient, and bias between the predicted and observed motor outcome scores. The approach was compared to individual networks given different data subsets as inputs.

**Results:**

The ensemble approach yielded a MAPE of 18.36%, MAE of 4.70, a Pearson’s correlation coefficient of 0.84, and had no significant bias indicating accurate outcome prediction. The approach outperformed individual networks not given DaTscan imaging or clinical measures of motor symptoms as inputs, respectively.

**Conclusion:**

The approach showed promise for longitudinal prognostication in PD and demonstrated the synergy of imaging and non-imaging information for the prediction task.

**Supplementary Information:**

The online version contains supplementary material available at 10.1186/s13550-021-00795-6.

## Background

Parkinson’s disease (PD) is one of the most common neurodegenerative disorders that is estimated to affect 10 million individuals globally [1, 2]. PD is characterized by the loss of striatal dopaminergic neurons in the substantia nigra and by progressive motor and non-motor symptoms, including bradykinesia, resting tremor, muscular rigidity, postural instability, and cognitive problems [1, 3, 4]. Diagnosis of PD is informed by imaging the dopamine transporter with [^123^I]ioflupane (DaTscan), an agent for dopamine transporter single-photon emission computed tomography [3].

Identifying biomarkers for PD progression and prediction of outcome in PD is an important clinical need [5, 6]. For this purpose, the Parkinson’s Progression Markers Initiative (PPMI) made available a longitudinal database of DaTscan images and clinical measures of patients with PD [7]. Deep learning methods based on convolutional neural networks (CNNs) and recurrent neural networks have had success in medical image classification and time series prediction, respectively [8, 9]. While deep learning methods can suffer from high variance in prediction [10], ensemble learning methods can improve accuracy of prediction by combining multiple classifier systems [11]. Several research groups have developed classifiers using machine learning, deep learning, and ensemble methods with traditional machine learning on PPMI data [5, 12–17]. For instance, Tang et al. used an artificial neural network to predict PD outcome as a binary classification task using radiomic features from DaTscan imaging [18]. However, using ensemble deep learning methods for building predictive models in PD with DaTscan imaging and non-imaging information has not been explored.

**Fig. 1:**
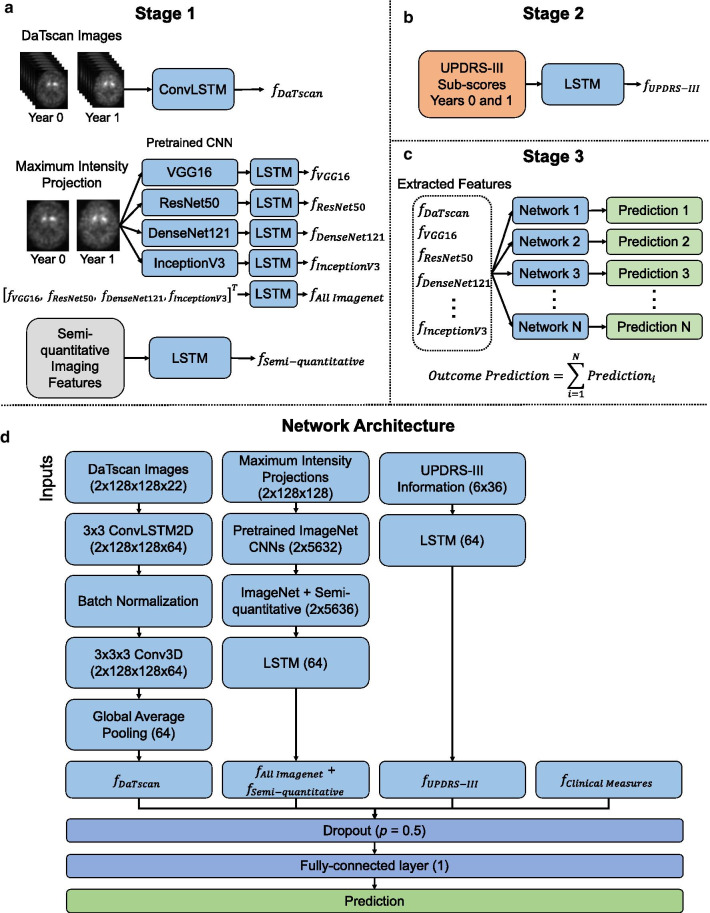
An illustration of the three-stage ensemble deep learning approach (**a**–**c**) and the complete network architecture with all inputs (**d**)

Our objective was to develop a three-stage, deep learning, ensemble approach for prognosis in patients with PD (Fig. 1). The approach was developed to predict longitudinal motor scores 4 years after initial baseline screening (Year 4) by incorporating imaging and non-imaging measures from baseline (Year 0) and 1 year after baseline (Year 1). The first stage extracted relevant spatiotemporal features directly from DaTscan imaging using a convolutional recurrent neural network architecture. The second stage extracted relevant temporal features from clinical motor scores using a recurrent neural network architecture to account for the time-series nature of the longitudinal motor scores. The third stage employed an ensemble learning approach that combined those extracted features and additional clinical measures to predict motor outcome of patients with PD in Year 4. The ensemble approach proved promising for prediction of motor outcome in patients with PD, provided multiple methods for extracting the relevant features from clinical data, and demonstrated synergy when combining imaging and non-imaging clinical measures for prediction.

## Methods

Retrospective data were obtained from the publicly available PPMI database (https://www.ppmi-info.org/) [7]. This study was reviewed by the Johns Hopkins Institutional Review Boards (IRB) and acknowledged as non-human subject research since deidentified pre-existing open-access data were used. From the PPMI database, 198 patients with PD (144 men, 54 women, mean age 67.60 ± 9.96 years, age range 39–91) with available DaTscan images at Years 0 and 1 were selected. Striatal binding ratio values in the left and right caudate nuclei and putamina of DaTscan images, referred to as semi-quantitative imaging features, were extracted [19]. Other clinical measures included age, gender, and duration of illness with respect to time at diagnosis and time of appearance of symptoms. The Movement Disorder Society Unified Parkinson’s Disease Rating Scale part III (MDS-UPDRS-III) scores, one of the most commonly used clinical rating scales for motor symptoms of PD, were extracted from Years 0, 1, and 4 [3, 7]. Data from Years 0 and 1 were used as predictors. Observed clinical MDS-UPDRS-III scores in Year 4 (mean 30.65 ± 10.53, range 9.33–77.00) were used as ground truth for the regression task where higher scores indicate more severe motor symptoms. Further details on data processing are provided in Additional file 1.

### Three-stage, deep learning, ensemble approach

The three-stage ensemble approach is illustrated in Fig. 1. Stage 1 extracted spatiotemporal features from baseline DaTscan images with three different methods (Fig. 1a). First, a convolutional long short-term memory (LSTM) network, a type of recurrent neural network, extracted features from DaTscan image volumes containing the complete structure of the striatum [20]. Second, CNNs pre-trained on the ImageNet dataset of natural images [21], including VGG16 [22], ResNet50 [23], DenseNet121 [24], and InceptionV3 [25], followed by an LSTM network extracted features from maximum intensity projections (MIPs) of DaTscan transaxial image slices. However, one constraint for such pre-trained CNNs is that the inputs are limited to 2D images. Therefore, MIPs of the DaTscan images were used as inputs to retain 3D information about the imaged volume. Third, an LSTM network extracted features from semi-quantitative imaging measures. See Additional file 1 for a detailed description of the DaTscan feature extraction methods.

Stage 2 extracted temporal features from baseline MDS-UPDRS-III time-sequences using an LSTM network (Fig. 1b). Stage 3 combined the extracted features from Stages 1 and 2 with other non-imaging clinical measures (age, gender, duration of illness) and placed those features into a fully connected layer to yield a prediction (Fig. 1c, d). Batch normalization and dropout with a drop probability of 0.5 were used to regularize the network [9]. Eleven networks were trained with different subsets of extracted DaTscan imaging features (Table 1). Inputs to all networks included clinical measures and MDS-UPDRS-III information. All network predictions were averaged in an ensemble to give the final predicted MDS-UPDRS-III score in Year 4.

**Table 1:** The different sets of imaging feature combinations used as input to the approach

	Feature set combinations
1	DaTscan + Semi-quantitative + All ImageNet
2	DaTscan + Semi-quantitative
3	DaTscan + All ImageNet
4	Semi-quantitative + All ImageNet
5	DaTscan
6	Semi-quantitative
7	All ImageNet
8	VGG16
9	ResNet50
10	DenseNet121
11	InceptionV3

DaTscan imaging features refer to the imaging features extracted directly from the convolutional LSTM network. Semi-quantitative refers to the striatal binding ratio values from the right and left caudate nuclei and putamina

### Training and evaluation

Data were randomly partitioned into training, validation, and test sets with 118, 40, and 40 patients, respectively, using a 60%/20%/20% split. Grid search hyperparameter optimization and training were performed using the training and validation sets. Model parameters were randomly initialized, and the approach was trained for 200 epochs with a batch size of 32. The model parameters of the CNNs pre-trained on ImageNet were frozen during training. The approach was trained with a mean absolute error (MAE) loss function that quantified the error between the observed and predicted MDS-UPDRS-III scores in Year 4 and the Adam optimization algorithm [26].

The approach was evaluated on the test set by assessing the mean absolute percentage error (MAPE), MAE, mean squared error (MSE), Pearson’s correlation coefficient, and an ordinary least squares linear regression between the predicted and observed MDS-UPDRS-III scores in Year 4 [27–30] (Additional file 1: Equations 1–5). The 95% confidence interval (CI), 95% prediction interval, and goodness-of-fit *R*^2^ value of the regression line were computed [31]. Scatter plots of the predicted versus observed MDS-UPDRS-III scores were created. Bland–Altman plot analysis was performed to evaluate bias and agreement between the observed and predicted outcome scores [32]. Bias was estimated by the mean difference between the observed and predicted outcome scores. The limits of agreement were estimated by calculating the mean difference ± 1.96*s*, where* s* is the standard deviation of the differences [32]. Failure analysis was performed for test cases with a MAPE greater than two standard deviations above the mean. See Additional file 1 for further details on training and evaluation.

### Varying the input data

To evaluate the approach’s robustness to different inputs, four individual networks trained on different subsets of the clinical inputs (Table 2) were compared to the ensemble approach, which used a combination of multiple trained networks. The first network was trained once on all available inputs. Clinical measures (age, gender, duration of illness), DaTscan imaging, and MDS-UPDRS-III information were excluded from the inputs for the second, third, and fourth networks, respectively. In all cases, the DaTscan imaging inputs refer to features extracted from DaTscan images using a convolutional LSTM network. Each network was evaluated with metrics described above and compared to the ensemble approach by evaluating the difference of squared errors (Additional file 1: Equation 6).

**Table 2:** The different subsets of feature combinations used as input

	Feature set combinations
1	DaTscan + MDS-UPDRS-III + Clinical Information
2	DaTscan + MDS-UPDRS-III (No Clinical Information)
3	MDS-UPDRS-III + Clinical (No DaTscan Information)
4	DaTscan + Clinical (No MDS-UPDRS-III Information)

DaTscan refers to the imaging features extracted directly from the convolutional LSTM network. Clinical information refers to the clinical measures of age, gender, and duration of illness with respect to time of diagnosis and time of appearance of symptoms

### Comparison of DaTscan feature extraction methods

To evaluate the image feature extraction methods, the 11 individual networks trained in Stage 1 with different subsets of imaging features (Table 1) were compared to the ensemble approach. VGG16, ResNet50, DenseNet121, and InceptionV3 refer to imaging features extracted from each CNN pre-trained on ImageNet separately (Table 1). Imaging features extracted from all four pre-trained CNNs were also combined and are referred to as All ImageNet imaging features (Table 1). Additional inputs to the network included MDS-UPDRS-III and clinical measures for each case.

### Statistical analysis

Statistical analysis was performed in Python 3.7.9. Statistical significance was present when *P* < 0.05. Statistically significant differences were determined using a paired two-tailed *t*-test. The normality of the predicted and observed MDS-UPDRS-III scores and their differences was confirmed by the Shapiro–Wilk test [33]. A one-sample *t*-test of the mean differences was performed where the null hypothesis that the true mean of differences is zero, corresponding to no bias, was tested. This was done to determine if there was a statistically significant bias between the observed and predicted MDS-UPDRS-III scores [34]. Experiments were implemented with TensorFlow 1.13.1 and Keras 2.2.5 and run on an NVIDIA Quadro P5000 GPU and Linux CentOS 7.6 operating system.

## Results

### Evaluating the ensemble approach

**Fig. 2:**
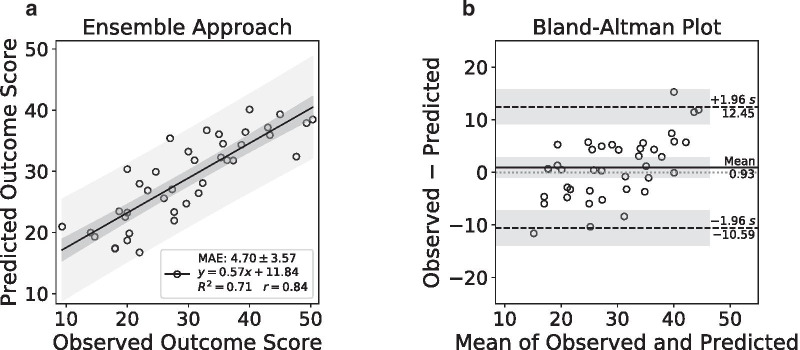
Performance of the ensemble approach on the test set. A scatter plot of the predicted versus observed outcome scores in Year 4 (**a**). A Bland–Altman plot of the differences versus the means of the observed and predicted outcome scores (**b**)

The three-stage, deep learning, ensemble approach yielded a MAPE of 18.36% (95% CI 11.74%, 24.98%), MAE of 4.70 (95% CI 3.56, 5.84), and MSE of 34.53 (95% CI 18.81, 50.25) between the predicted and observed MDS-UPDRS-III scores on the test set (Table 3). The approach also yielded a Pearson’s correlation coefficient of 0.84 (*P* < 0.001) on the test set, indicating a strong positive correlation between the predicted and observed MDS-UPDRS-III scores. Figure 2a depicts a scatter plot of the predicted versus observed MDS-UPDRS-III scores. The regression line computed by ordinary least squares regression and the corresponding 95% confidence and prediction intervals, shown by the dark and light gray shaded regions, respectively, were overlaid on the scatter plot in Fig. 2a. The *R*^2^ value for the regression line for the ensemble approach was 0.71, indicating a strong relationship between the predicted and observed MDS-UPDRS-III scores in Year 4.

**Table 3:** Varying the input data

Method	MAPE	MAE	MSE	*r*	*R*^2^
Ensemble approach	18.36% (11.74%, 24.98%)	4.70 (3.56, 5.84)	34.53 (18.81, 50.25)	0.84	0.71
DaTscan + MDS-UPDRS-III + Clinical Information	19.89% (12.48%, 27.30%)	5.04 (3.78, 6.29)	40.41 (22.09, 58.74)	0.81	0.66
DaTscan + MDS-UPDRS-III (No Clinical Information)	19.89% (13.63%, 26.15%)	5.22 (4.09, 6.35)	39.37 (24.48, 54.25)	0.81	0.66
MDS-UPDRS-III + Clinical (No DaTscan Information)	26.33% (17.76%, 34.91%)	6.63 (5.11, 8.14)	65.85 (36.14, 95.57)	0.64	0.41
DaTscan + Clinical (No MDS-UPDRS-III Information)	35.48% (22.50%, 48.46%)	9.15 (6.90, 11.39)	131.71 (73.60, 189.81)	0.04 (n.s.)	0.00

Data in parentheses are 95% confidence intervals

MAPE, mean absolute percentage error; MAE, mean absolute error; MSE, mean squared error; n.s., not significant; *r*, Pearson correlation coefficient; *R*^2^, coefficient of determination. Unless indicated, values for *r* were significant (*P* < 0.001)

Figure 2b depicts a Bland–Altman plot of the differences versus the means between the observed and predicted MDS-UPDRS-III scores. The ensemble approach had a mean difference of 0.93 (95% CI − 0.95, 2.81) shown by the solid horizontal line. The limits of agreement were from − 10.59 (95% CI − 13.84, − 7.33) to 12.45 (95% CI 9.19, 15.70) shown by the dashed horizontal lines. The corresponding 95% confidence intervals shown by the shaded regions were overlaid on the Bland–Altman plot (Fig. 2b). A one-sample *t*-test of the mean differences confirmed that there was no evidence of bias (*P* = 0.32) between the observed and predicted MDS-UPDRS-III scores in Year 4 by the ensemble approach.

There was only one case (age 73 years, gender man) from the test set (1/40) that had a MAPE of greater than two standard deviations above the mean (MAPE greater than 59.25%). For this case, the approach had a MAPE of 124.46%, MAE of 11.62, and MSE of 134.93. The observed and predicted MDS-UPDRS-III scores for this patient were 9.33 and 20.95, respectively.

### Varying the input data

**Fig. 3:**
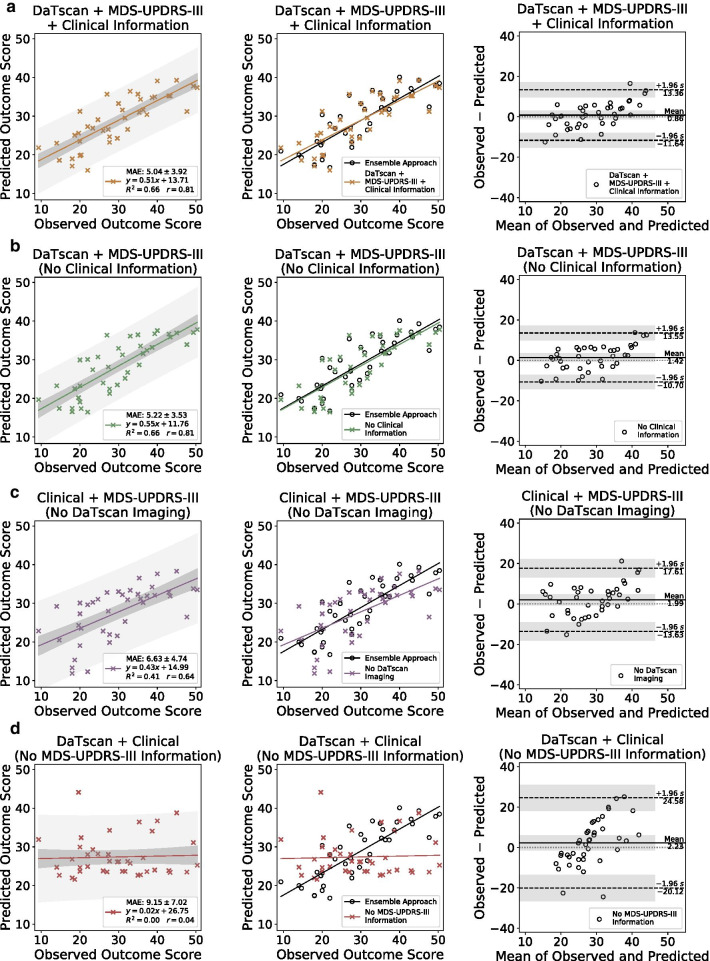
Scatter plots of the predicted versus observed MDS-UPDRS-III scores, comparisons with the ensemble approach, and Bland–Altman plots on the test set by the networks trained with different input feature combinations are shown in each column from left to right, respectively

The performances of the networks trained with different subsets of input features (Table 2) were evaluated and compared to the ensemble approach that was trained with all of the available input features. Performance metrics for each network are summarized in Table 3. Scatter plots of the predicted versus observed MDS-UPDRS-III scores in Year 4 as predicted by the networks trained with different subsets of input features are shown for each case in Fig. 3. The performance of the ensemble approach was compared to each case by overlaying the scatter plot of the predicted versus observed MDS-UPDRS-III scores. Regression lines and the corresponding 95% confidence and prediction intervals computed by ordinary least squares regression are also shown. Bland–Altman plots with the mean differences and limits of agreement are shown for each case in Fig. 3. There was no evidence of significant bias in each case by a one-sample *t*-test of mean differences (*P* > 0.05).

The ensemble approach significantly outperformed the individual network trained on all available inputs (Fig. 3a), on the basis of MSE (*P* < 0.05). The ensemble approach significantly outperformed the network trained on inputs that excluded clinical measures of age, gender, and duration of illness (Fig. 3b), on the basis of MAE (*P* < 0.05). The ensemble approach also significantly outperformed the network trained on inputs that excluded DaTscan imaging information (Fig. 3c) and the network trained on inputs that excluded MDS-UPDRS-III information (Fig. 3d), on the basis of MAPE, MAE, and MSE (*P* < 0.05). The ensemble approach had the highest Pearson’s correlation coefficient (0.84) and *R*^2^ value (0.71) when compared to the other networks that were given varying input feature sets (Table 3), indicating a more accurate prediction.

The performances of the networks that were not given DaTscan imaging and MDS-UPDRS-III information as inputs were significantly reduced (*P* < 0.05) when compared to the performance of the network that received all the training inputs, on the basis of MAPE, MAE, and MSE (Table 3). The performance of the network that was only trained on DaTscan imaging and MDS-UPDRS-III information (inputs excluded clinical measures of age, gender, duration of illness) also significantly outperformed the networks that were not given DaTscan imaging or MDS-UPDRS-III information as inputs, respectively, on the basis of MAPE, MAE, and MSE (Table 3). The two networks that were given at minimum both DaTscan and MDS-UPDRS-III information as inputs (Fig. 3a, b) both yielded a Pearson’s correlation coefficient greater than 0.80 and an *R*^*2*^ value of 0.66, indicating relatively high performance on the outcome prediction task.

The network that was not given MDS-UPDRS-III information as inputs had the largest mean difference of 2.23 (95% CI − 1.42, 5.88) and the widest limits of agreement from − 20.12 to 24.58 by Bland–Altman analysis (Fig. 3d). The network that was not given DaTscan inputs had the second-largest mean difference of 1.99 (95% CI − 0.56, 4.54) and the second widest limits of agreement from − 13.63 to 17.61 (Fig. 3c). In comparison, the network that was given all the inputs (Fig. 3a) and the network that was given at both DaTscan and MDS-UPDRS-III inputs (Fig. 3b) had smaller mean differences (0.86 (95% CI − 1.18, 2.90) and 1.42 (95% CI − 0.55, 3.40), respectively) and tighter limits of agreement (− 11.64 to 13.36 and − 10.70 to 13.55, respectively).

For the network not given MDS-UPDRS-III inputs, there is a positive correlation between the differences and the means of the observed and predicted outcome scores by visual inspection of the Bland–Altman plot (Fig. 3d). This suggests that the network in this case tends to overestimate outcome scores for subjects with lower observed scores and underestimate those with higher scores. A similar trend can be seen for the network not given DaTscan inputs to a lesser extent (Fig. 3c). This effect is greatly reduced in the cases of the two networks that were given at least both DaTscan and MDS-UPDRS-III inputs (Fig. 3a, b).

**Fig. 4:**
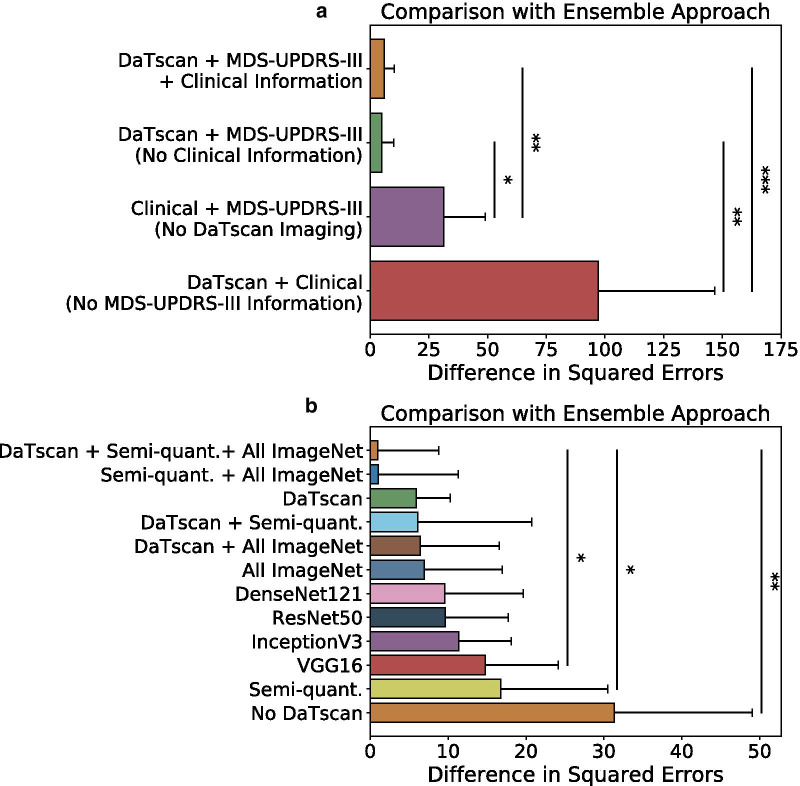
Comparison of the ensemble approach to the individual networks that were given different subsets of inputs (**a**) and different subsets of imaging features (**b**). *P < 0.05; **P < 0.005; and ***P < 0.0005

Figure 4a shows the performance measured by the difference in squared errors of the networks trained with different subsets of input features as compared to the ensemble approach. The network that was trained with all available inputs had a difference in squared errors of 5.89 (95% CI 1.51, 10.27) and significantly outperformed the networks that were not given baseline DaTscan imaging and MDS-UPDRS-III information as inputs, respectively, on the basis of difference in squared errors (*P* < 0.05) (Fig. 4a). The network that was trained with only DaTscan and MDS-UPDRS-III information (inputs excluded other clinical measures) significantly outperformed the networks that were not given DaTscan imaging or MDS-UPDRS-III information as inputs, respectively, on the basis of difference in squared errors (*P* < 0.05) (Fig. 4a).

### Comparison of DaTscan feature extraction methods

**Fig. 5:**
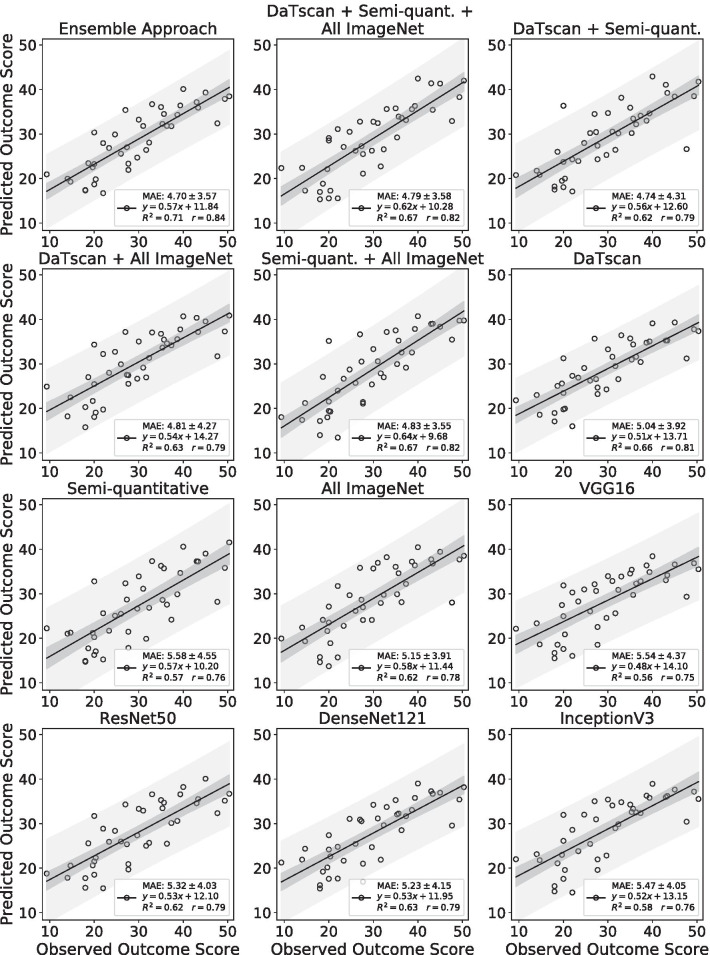
Scatter plots with regression lines of the predicted versus observed outcome scores in Year 4 on the test set by the networks trained with different input imaging feature combinations given in Table 1

**Fig. 6:**
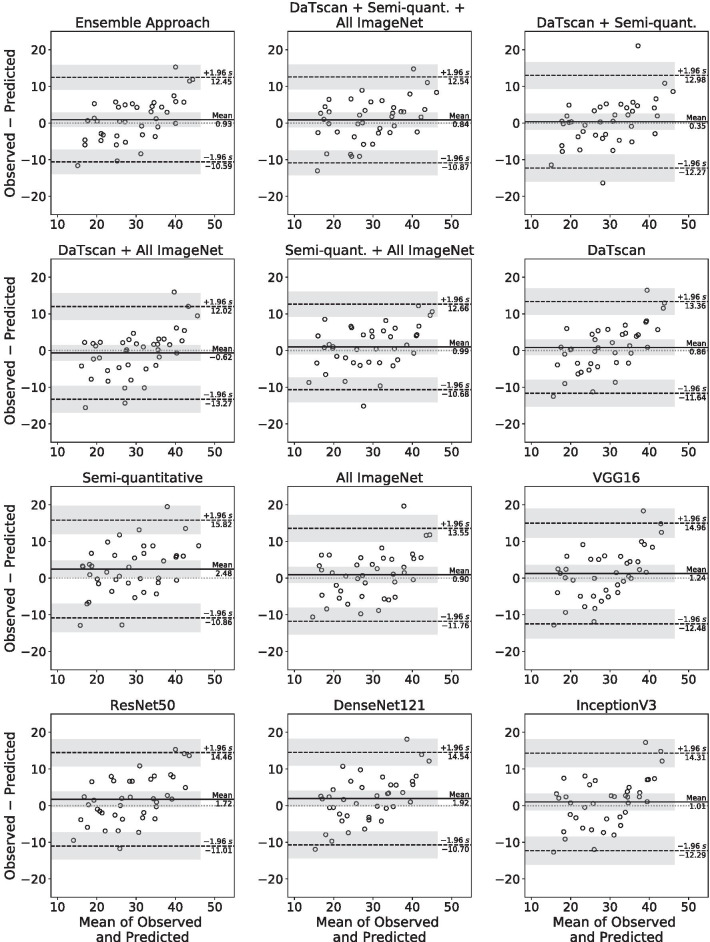
Bland–Altman plots of the differences versus the means between the predicted and observed outcome scores in Year 4 on the test set by the networks trained with different input imaging feature combinations given in Table 1

The performance of the ensemble approach was compared to 11 networks each trained with different subsets of input imaging features (Table 1) in addition to baseline MDS-UPDRS-III information and other clinical measures. The performances of those networks were evaluated on the basis of standard evaluation metrics and are summarized in Table 4. The ensemble approach had the lowest MAPE, MAE, and MSE compared to networks trained with different subsets of input imaging features. The ensemble approach also had the highest Pearson’s correlation coefficient and *R*^2^ value when compared to other cases, indicating higher accuracy in the prediction task. Scatter plots of the predicted versus observed MDS-UPDRS-III scores and the corresponding regression lines for each case were created (Fig. 5). Bland–Altman plots with the corresponding mean differences and limits of agreement were also created for each case (Fig. 6).

**Table 4:** Comparison of DaTscan image feature extraction methods

Method	MAPE	MAE	MSE	*r*	*R*^2^
Ensemble approach	18.36% (11.74%, 24.98%)	4.70 (3.56, 5.84)	34.53 (18.81, 50.25)	0.84	0.71
DaTscan + Semi-quantitative + All ImageNet	19.64% (12.09%, 27.18%)	4.79 (3.65, 5.94)	35.48 (20.11, 50.85)	0.82	0.67
DaTscan + Semi-quantitative	18.82% (11.32%, 26.31%)	4.74 (3.36, 6.12)	40.60 (14.59, 66.60)	0.79	0.62
DaTscan + All ImageNet	20.64% (11.50%, 29.79%)	4.81 (3.45, 6.18)	40.95 (19.94, 61.96)	0.79	0.63
Semi-quantitative + All ImageNet	18.82% (12.75%, 24.89%)	4.83 (3.69, 5.96)	35.57 (20.41, 50.73)	0.82	0.67
DaTscan	19.89% (12.48%, 27.30%)	5.04 (3.78, 6.29)	40.41 (22.09, 58.74)	0.81	0.66
Semi-quantitative	21.43% (13.76%, 29.09%)	5.58 (4.13, 7.03)	51.29 (27.25, 75.33)	0.76	0.57
All ImageNet	20.18% (13.76%, 26.59%)	5.15 (3.90, 6.40)	41.44 (20.15, 62.72)	0.78	0.62
VGG16	21.67% (13.99%, 29.35%)	5.54 (4.14, 6.94)	49.31 (26.95, 71.67)	0.75	0.56
ResNet50	19.70% (13.82%, 25.58%)	5.32 (4.03, 6.61)	44.14 (25.64, 62.65)	0.79	0.62
DenseNet121	20.24% (13.04%, 27.45%)	5.23 (3.90, 6.56)	44.12 (23.23, 65.01)	0.79	0.63
InceptionV3	21.89% (14.30%, 29.48%)	5.47 (4.17, 6.76)	45.89 (25.32, 66.46)	0.76	0.58

Data in parentheses are 95% confidence intervals

All values for r were significant (*P* < 0.001)

MAPE, mean absolute percentage error; MAE, mean absolute error; MSE, mean squared error; *r*, Pearson correlation coefficient; *R*^2^, coefficient of determination

The performances of those networks were compared to the ensemble approach by computing the difference in squared errors (Fig. 4b). The network that was trained with all available imaging features derived from DaTscan imaging, semi-quantitative imaging measures, and All ImageNet imaging features had the best relative performance and yielded the lowest difference in squared errors of 0.95 (95% CI − 6.89, 8.80). The network trained on all available imaging features also significantly outperformed the networks that were trained with only VGG16 features and semi-quantitative imaging features (*P* < 0.05), respectively, on the basis of the difference in squared errors (Fig. 4b).

There was no evidence of significant bias for all networks trained with different input imaging features (*P* > 0.05) by Bland–Altman analysis, with the exception of the network that was given only semi-quantitative imaging features as inputs which had a significant bias (*P* < 0.05) with a mean difference of 2.48 (95% CI 0.30, 4.65) (Fig. 6). All networks trained with different imaging features, except for the network trained only with semi-quantitative imaging features, significantly outperformed the network that was not given any DaTscan imaging features as inputs on the basis of MAE, MSE, and the difference in squared errors (*P* < 0.05).

## Discussion

A three-stage, deep learning, ensemble approach was developed for longitudinal outcome prediction of patients with PD. The approach took DaTscan imaging, MDS-UPDRS-III information, and other clinical measures, including age, gender, and duration of illness, from Years 0 and 1 as inputs and trained multiple neural networks to extract relevant features to accurately predict outcomes in Year 4. The ensemble approach outperformed networks that were not given DaTscan imaging or MDS-UPDRS-III information demonstrating the importance of combining imaging and clinical measures for the outcome prediction task. The approach provided multiple methods for extracting features from DaTscan images and showed improved performance when all sources of extracted imaging features were used as inputs to the network.

The approach was studied in the context of varying the training inputs to the network. The networks that were not given MDS-UPDRS-III or DaTscan information as inputs had the largest and second-largest reduction in performance across all evaluation metrics, respectively, when compared to the network that received all of the training inputs (Fig. 4a). That emphasizes the relative importance of DaTscan imaging and MDS-UPDRS-III information for the outcome prediction task. While this suggests that MDS-UPDRS-III information from Years 0 and 1 was most important for the prediction task, DaTscan imaging features also contributed significantly to the performance of the approach.

The performance of the approach was also evaluated in the context of training 11 networks each with a different subset of the extracted input imaging features (Stage 1). The ensemble approach had higher performance than that of the 11 individual networks across several standard evaluation metrics, highlighting the utility of the ensemble learning approach. The network that received information from all available imaging measures, including the DaTscan images, semi-quantitative imaging measures, and imaging features extracted from the CNNs pre-trained on ImageNet, had the highest relative performance on the basis of the difference of squared errors when compared to the ensemble approach.

Networks that received two or more sources of DaTscan imaging input features tended to perform better than those that received only one source of extracted imaging features. Four of the top five performers, on the basis of difference in squared errors, were given some combination of the DaTscan images, semi-quantitative imaging measures, or features extracted from CNNs pre-trained on ImageNet as inputs (Fig. 4b). This suggests that complementary information relevant for the prediction task was extracted from the different sources of DaTscan imaging information. The networks that received at least one source of DaTscan imaging input features, except for the network trained only on semi-quantitative imaging features, significantly outperformed the network that was not given any DaTscan information (*P* < 0.05), emphasizing the importance of DaTscan imaging for the prediction task.

In a previous study, the performance of four motor performance measures, including functional reach, timed hall walk, timed block sort task, and timed dotting, was evaluated for predicting outcomes in PD [6]. Correlation values ranging from 0.29 to 0.49 were observed between those motor performance measures and MDS-UPDRS-III motor scores in patients with PD [6]. In contrast, the ensemble approach achieved a Pearson’s correlation of 0.84 on the test set, outperforming those motor performance measures on the prognosis task. Previous studies predicted disease progression only up to 2 years after follow-up [6, 13], whereas our approach performed prognosis of patients with PD 4 years after the initial baseline scan, highlighting the utility of the approach. Additionally, the approach extracted information from DaTscan images directly without the need for performing co-registration, segmentation, or defining regions of interest on the corresponding magnetic resonance images as done in other studies [5, 18, 35].

The ensemble approach proved promising for the prediction of outcomes in patients with PD. The approach may be incorporated into a prognostic tool to characterize patients with PD into different groups based on disease progression. Such a prognostic tool may facilitate therapy—palliative or disease-modifying, when available—tailored to an individual patient’s needs. It can also be used to educate the patient and his/her family regarding likely outcomes.

The clinical dataset used in this study for training, validation, and testing consisted of 198 patients from the PPMI database. However, deep learning methods usually require very large training data sizes, on the order of thousands, to adequately train deep neural networks [9]. To address the issue of a limited clinical dataset, we extracted features from DaTscan images with four commonly used CNN architectures that were pre-trained on the ImageNet dataset, consisting of millions of natural images across 1000 different class label categories. This was done to extract generalized spatial features from DaTscan images. Indeed, the two networks with the highest performance, on the basis of difference in squared errors, were given imaging features extracted from CNNs pre-trained on ImageNet as inputs (Fig. 4b). An alternative approach to combat the limited amount of clinical data would be to generate a large amount of simulated training data to train the approach. For example, a physics-guided simulation-based framework was developed to improve the performance of a deep learning model on segmenting lung cancer lesions [36]. Generative adversarial networks could also be used to generate a large amount of simulated data to train the approach [37]. Incorporating such simulation-based training strategies could further improve the performance of the ensemble approach.

External validation of the approach using clinical data from different sites and scanners outside of the PPMI database is also important to evaluate the performance of the approach in clinical scenarios. Such external validation would require the collection of longitudinal clinical data from patients with Parkinson’s disease in a prospective study. While this is outside the scope of the present study, this is an important area of further research. Validation of the approach using DaTscan imaging data generated with different reconstruction algorithms is also an important issue to assess the clinical applicability of the approach. For instance, quantitative reconstruction methods can improve the accuracy of striatal binding potential estimates by providing DaTscan images with improved contrast and resolution [38]. Training the approach on data reconstructed with such quantitative reconstruction methods could improve the performance of the approach.

The approach can be trained in a bootstrap aggregating fashion where each network comprising the ensemble is trained in parallel [11]. However, larger computational resources are required compared to training a single model, which may limit the utility of the approach in a clinical setting where such resources may not be available. The single network that was trained on all of the available imaging features also had a relatively high performance on the prognostic task and may be acceptable for use in such cases. We focused on baseline DaTscan imaging and MDS-UPDRS-III subscores that reflect motor symptoms as inputs for the prediction task. Extending the approach to incorporate additional neuroimaging genetic information as well as clinical measures that reflect non-motor symptoms, such as mood or cognitive function, as inputs may further improve performance [13, 35, 39]. The approach employed ensemble averaging where all model outputs were averaged to yield the final prediction and showed improved performance over individual models. Notably, Xiao et al. developed a meta-learner method for cancer detection based on stacked generalization where the outputs of machine learning classifiers were used as inputs into another neural network that performed the final classification [40]. Integrating the approach in such a meta-learner scheme could further improve performance.

## Conclusion

A three-stage, deep learning, ensemble approach was developed and provided accurate prediction of motor outcome in Year 4 using baseline data. The approach provided several methods for extracting relevant spatiotemporal imaging features from DaTscan images and demonstrated the capacity of synergy between imaging and non-imaging information for refining prediction.

## Supplementary Information

**Additional file 1**. Supplemental methods.

## Data Availability

Data in this study were obtained from the publicly available Parkinson’s Progression Markers Initiative (PPMI) database (https://www.ppmi-info.org/).

## Abbreviations

CI: Confidence interval
CNN: Convolutional neural network
DaTscan: [^123^I]ioflupane dopamine transporter single photon emission tomography
LSTM: Long short-term memory
MAE: Mean absolute error
MAPE: Mean absolute percentage error
MDS-UPDRS-III: Movement Disorder Society Unified Parkinson’s Disease Rating Scale part III
MIP: Maximum intensity projection
MSE: Mean squared error
PD: Parkinson’s disease
PPMI: Parkinson’s Progression Markers Initiative
