# Supplementary material for: A three-stage, deep learning, ensemble approach for prognosis in patients with Parkinson’s disease
Source: EJNMMI Res. 2021 Jun 7;11:52. doi: 10.1186/s13550-021-00795-6 (PMC8184905; doi:10.1186/s13550-021-00795-6)
Supplement: Supplementary file 1 — Additional file 1. Supplemental methods. [file 13550_2021_795_MOESM1_ESM.pdf]

A three-stage, deep learning, ensemble approach for prognosis in patients with Parkinson’s disease

Kevin H. Leung[1,2], Steven P. Rowe[2], Martin G. Pomper[1,2], Yong Du[2]

[1]Department of Biomedical Engineering, Johns Hopkins University School of Medicine, Baltimore, MD, USA

[2]The Russell H. Morgan Department of Radiology and Radiological Science, Johns Hopkins University School of Medicine, Baltimore, MD, USA

Corresponding Author: Kevin H. Leung

Email: kleung8@jhmi.edu

Address: 601 N Caroline St. JHOC 4263, Baltimore, MD 21287, USA.

EJNMMI Research

Supplemental Methods

Data processing

The DaTscan images were preprocessed by selecting a continuous segment of 22 axial image slices of each image volume where the central slice had the highest relative mean uptake intensity. This was done to capture the structure of the striatum and to remove image slices of relatively lower intensity and higher noise. The resulting DaTscan images had a cubic voxel size of 2 mm, were zero-padded to yield an image size of 128 × 128 × 22, and normalized to values from 0 to 1.

A time series of measured MDS-UPDRS-III subscores relating to motor signs of Parkinson's disease (PD) were extracted at the time points of screening, baseline, 3, 6, 9, 12, 42, 48, and 54 months. Those subscores reflected the motor signs of PD, including speech, facial expression, rigidity, finger tapping, hand movements, pronation-supination movements of hands, toe-tapping, leg agility, arising from a chair, gait, freezing of gait, postural stability, posture, body bradykinesia, postural and kinetic tremor of the hands, rest tremor amplitude, and constancy of rest tremor. Information about whether the patient was receiving medication for treating symptoms of PD and the clinical state of patients receiving medication (good or poor response) at each time-point were also extracted [1]. MDS-UPDRS-III scores missing at any time point were set to be equal to the value of the previous time step following the last observation carried forward imputation procedure. The observed MDS-UPDRS-III subscores, overall MDS-UPDRS-III score, and treatment information at Years 0 to 1 (screening, baseline, 3, 6, 9, and 12 months) were used as inputs to the approach. This resulted in an input time sequence consisting of six time-points (from screening to 12 months) with thirty-six features that are referred to as the input MDS-UPDRS-III information.

The MDS-UPDRS-III subscores at 42, 48, and 54 months were summed and averaged to yield the overall MDS-UPDRS-III scores at Year 4 which were used as outcome. The outcome

prediction task was formulated as a regression task since the overall MDS-UPDRS-III score at Year 4 is a continuous value.

Image feature extraction with a convolutional LSTM-based network architecture

The DaTscan images at Years 0 and 1 were input as a time sequence into a convolutional LSTM-based network architecture for feature extraction (Fig 1a). The convolutional LSTM network is a type of recurrent neural network architecture that is similar to an LSTM-based architecture where the input and recurrent transformations are both convolutional. The convolutional LSTM-based networks can better capture spatiotemporal correlations in the input data where the input data are spatiotemporal sequences [2].

The DaTscan image volumes at Years 0 and 1 consisted of 22 axial slices that contained the complete structure of the striatum at two time points. The output of the convolutional LSTM layer was then placed into a batch normalization layer followed by a three-dimensional (3D) convolutional layer and 3D global average pooling layers. Batch normalization has been shown to stabilize learning and accelerate training by normalizing each batch of inputs into subsequent layers of the network [3]. The output of the global average pooling layer was an N-dimensional extracted feature vector containing information about the original input DaTscan images from Years 0 and 1. Here, the dimensionality of the extracted feature vector was N=64.

Image feature extraction with pre-trained CNNs

Deep learning methods typically require very large training data sizes, on the order of thousands, to adequately train deep neural networks on various image analysis tasks [3]. Due to our limited dataset consisting of only 198 patients, we extracted features from DaTscan images at Years 0 and 1 with four commonly used CNN architectures that were pre-trained on the ImageNet dataset [4], including VGG16 [5], ResNet50 [6], DenseNet121 [7], and InceptionV3 [8]. The ImageNet dataset consists of millions of natural images across 1,000 different class label categories [4]. We hypothesized that these CNNs that were pre-trained on the natural image

classification task with the ImageNet dataset should be able to extract generalized spatial features from DaTscan images.

The maximum intensity projection (MIP) was first performed in the longitudinal direction of the DaTscan image slices (Fig 1). The MIPs obtained from the DaTscan images from Years 0 and 1 were used as input to the pre-trained VGG16, ResNet50, DenseNet121, and InceptionV3 CNN-based architectures. Since these pre-trained CNNs can only take 2D images as inputs, MIPs of the DaTscan images were used as inputs to the pre-trained networks instead of 3D image volumes. The MIPs were used to retain 3D information about the imaged volume. The CNN-based architectures were originally pre-trained on the image classification task on natural images from the ImageNet dataset. Imaging features were extracted from the last layer before the classification layer of each pre-trained network. These feature maps were input into a 2D global average pooling layer resulting in N-dimensional feature vectors containing information about the MIPs of DaTscan images from Years 0 and 1. The dimensionality of the feature vectors extracted from the VGG16, ResNet50, DenseNet121, and InceptionV3 networks were N=512, 2048, 1024, and 2048, respectively. Note that these pre-trained CNNs were not further trained or fine-tuned on the clinical data and were used simply as feature extractors.

The feature vectors corresponding to the MIPs from Years 0 and 1 were extracted from each pre-trained CNN-based architecture separately. The feature vectors were treated as a time sequence consisting of two timepoints at Years 0 and 1. This time sequence was then placed into an LSTM-based network architecture to capture the temporal features from the MIPs of DaTscan images at Years 0 and 1. The feature vectors extracted from each pre-trained CNN architecture were also combined into one feature vector with a dimensionality of N=5632 (Fig 1), which was referred to as the “All ImageNet” feature vector. The All ImageNet feature vector from Years 0 and 1 was also treated as a time sequence and was used as input to the LSTM-based network (Fig 1).

In summary, the relevant spatial features present in the DaTscan images were first extracted using the pre-trained CNN-based architectures. Those spatial features extracted from DaTscan imaging were then used as input to an LSTM network, which extracted the relevant temporal features [2]. This differs from the previous method where the relevant spatiotemporal features were extracted directly from the original DaTscan images using a convolutional LSTM-based architecture in one step.

Image feature extraction using semi-quantitative imaging measures

The semi-quantitative imaging measures of the striatal binding ratio of the left caudate, right caudate, left putamen and right putamen were also used as predictors for the prediction task. The striatal binding ratios were extracted from the PPMI database. The striatal binding ratio is defined as the ratio of specific uptake in the striatum to non-specific uptake in the background. Semi-quantitative imaging measures were input as a time sequence that consisted of two time-points at Years 0 and 1 to an LSTM network which extracted N-dimensional feature vectors corresponding to the relevant temporal features for the prediction task (Fig 1). Here, the dimensionality of the extracted feature vector was N=64.

Training and hyperparameter optimization

The approach was trained by optimizing a mean absolute error loss function that quantified the error between the measured and predicted MDS-UPDRS-III scores in Year 4. The network was optimized via a first-order gradient-based optimization algorithm, Adam [9]. A grid search was performed for hyperparameter optimization of the approach. The general range for each hyperparameter sweep spanned several orders of magnitude. The optimized hyperparameters included batch size, dropout probability, number of training epochs, and the dimensionality of the N-dimensional feature vectors extracted from baseline DaTscan imaging (Stage 1) and MDS-UPDRS-III subscores (Stage 2). Batch size is defined as the number of training examples used to update the network weights for each iteration of training. An epoch is defined as one pass over all the examples in the training set while training the network. The range of batch sizes tested was

4, 8, 16, 32, and 64. The range for dropout probability was 0, 0.3, 0.5, and 0.8. The range for the number of training epochs was 75, 100, 150, 200, 250, 300, 500, and 1,000. The range for the dimensionality of the N-dimensional extracted feature vectors was N=4, 8, 16, 32, 64, 128, and 256.

Hyperparameter optimization was performed by training the proposed approach on the training set for each combination of hyperparameter values via grid search. The best performing combination of hyperparameter values was considered to be the combination that yielded the smallest mean absolute error loss function value on the validation set. The optimal hyperparameters were found to be N=64 (dimensionality of feature vectors), batch size of 32, training epochs of 200, and dropout probability of 0.5. After the optimized hyperparameters were selected, the approach was trained on the data from the training and validation sets consisting of 158 patients using those hyperparameters.

Evaluation metrics

The approach was evaluated on the test set of 40 patients. The accuracy of the approach was quantified by evaluating several standard evaluation metrics, including mean absolute percentage error (MAPE), mean absolute error (MAE), mean squared error (MSE), and Pearson's correlation coefficient (*r*) [10–12].

The evaluation metrics of MAPE, MAE, and MSE quantify the error between the predicted and observed MDS-UPDRS-III scores in Year 4 for the regression task and are defined as in equations 1, 2, and 3, respectively.

$$Equ.(1) \qquad MAPE = \frac{1}{N}\sum_{i=1}^{N}\left|\frac{\hat{y}_i - y_i}{y_i}\right| \times 100$$

$$Equ.(2) \qquad MAE = \frac{1}{N}\sum_{i=1}^{N}|\hat{y}_i - y_i|$$

$$Equ.(3) \qquad MSE = \frac{1}{N}\sum_{i=1}^{N}(\hat{y}_i - y_i)^2$$

The term $\hat{y}_i$ is defined as the predicted MDS-UPDRS-III score, the term y_i is defined as the observed MDS-UPDRS-III score for the ith sample, and N is defined as the sample size. The vertical bars denote absolute value in equations 1 and 2. For metrics of MAPE, MAE, and MSE, lower values indicate a more accurate prediction of the MDS-UPDRS-III score in Year 4.

Pearson's correlation coefficient measures the linear correlation between the predicted and observed MDS-UPDRS-III scores in Year 4 and ranges from -1 to +1 where larger positive values indicate a larger positive correlation and vice versa for negative values. Higher values of the Pearson's correlation coefficient between the predicted and observed MDS-UPDRS-III scores in Year 4 indicate more accurate prediction. As a rule of thumb, correlation coefficient values greater than 0.7 indicate a high positive correlation [12]. The Pearson's correlation coefficient is defined in equation 4 where cov is defined as covariance and σ is defined as the standard deviation [12].

$$Equ. (4) \qquad r = \frac{cov(\hat{y}_i, y_i)}{\sigma_{\hat{y}_i}\sigma_{y_i}}$$

To further evaluate the performance of the proposed approach, an ordinary least squares linear regression [13] was performed between the predicted and observed MDS-UPDRS-III scores in Year 4. The ordinary least squares regression fit a linear model solving for the intercept (β_1) and slope (β_2) in equation 5 that best fits the relationship between the predicted and observed MDS-UPDRS-III scores.

$$Equ. (5) \qquad \hat{y}_i = \beta_1 + \beta_2 y_i$$

The coefficient of determination or R^2 value which indicates the goodness-of-fit of the regression [14] was reported as an evaluation metric for the approach. The coefficient of determination indicates the amount of the total variance in the data that is explained by the fitted linear model. Values for R^2 range from 0 to 1 where higher values of R^2 indicate a more accurate prediction of the MDS-UPDRS-III score in Year 4. An R^2 value greater than 0.7 generally indicates a strong relationship between the observed data and the fitted values.

The approach was compared to cases where a single network was given different subsets of the clinical data as inputs. The difference of squared errors given by equation 6 was used to compare the performance of the approach to that of those networks.

$$Equ.(6) \qquad MSE_{Diff,j} = \frac{1}{N}\sum_{i=1}^{N}\left[\left(\hat{y}_{i,j} - y_i\right)^2 - \left(\hat{y}_{i,ensemble} - y_i\right)^2\right]$$

The term $\hat{y}_{i,j}$ is defined as the predicted MDS-UPDRS-III score in Year 4 for the ith sample by the network trained using the feature subset combination for the jth case for $j = 1, 2, 3, \ldots$ (Tables 1 and 2). The term $\hat{y}_{i,ensemble}$ is defined as the predicted MDS-UPDRS-III score in Year 4 for the ith sample by the ensemble approach. Positive values for the difference of squared errors indicate relatively worse performance in each case when compared to the performance of the ensemble approach and vice versa for negative values. Lesser values indicate a more accurate prediction of the MDS-UPDRS-III scores in Year 4 when compared to the ensemble approach.

Supplemental References

1. Marek K, Jennings D, Lasch S, Siderowf A, Tanner C, Simuni T, et al. The parkinson progression marker initiative (PPMI). Prog Neurobiol. Elsevier; 2011;95:629–35.

2. Xingjian SHI, Chen Z, Wang H, Yeung D-Y, Wong W-K, Woo W. Convolutional LSTM network: A machine learning approach for precipitation nowcasting. Adv Neural Inf Process Syst. 2015. p. 802–10.

3. Shen D, Wu G, Suk H-I. Deep learning in medical image analysis. Annu Rev Biomed Eng. Annual Reviews; 2017;19:221–48.

4. Deng J, Dong W, Socher R, Li L-J, Li K, Fei-Fei L. Imagenet: A large-scale hierarchical image database. 2009 IEEE Conf Comput Vis pattern Recognit. Ieee; 2009. p. 248–55.

5. Simonyan K, Zisserman A. Very deep convolutional networks for large-scale image recognition. arXiv Prepr arXiv14091556. 2014;

6. He K, Zhang X, Ren S, Sun J. Deep residual learning for image recognition. Proc IEEE Conf Comput Vis pattern Recognit. 2016. p. 770–8.

7. Huang G, Liu Z, Van Der Maaten L, Weinberger KQ. Densely connected convolutional networks. Proc IEEE Conf Comput Vis pattern Recognit. 2017. p. 4700–8.

8. Szegedy C, Vanhoucke V, Ioffe S, Shlens J, Wojna Z. Rethinking the inception architecture for computer vision. Proc IEEE Conf Comput Vis pattern Recognit. 2016. p. 2818–26.

9. Kingma D, Ba J. Adam: A Method for Stochastic Optimization. Int. Conf. Learn. Represent. 2014.

10. De Myttenaere A, Golden B, Le Grand B, Rossi F. Mean absolute percentage error for regression models. Neurocomputing. Elsevier; 2016;192:38–48.

11. Wang Z, Bovik AC. Mean squared error: Love it or leave it? A new look at signal fidelity measures. IEEE Signal Process Mag. IEEE; 2009;26:98–117.

12. Mukaka MM. A guide to appropriate use of correlation coefficient in medical research. Malawi Med J. 2012;24:69–71.

13. Kilmer JT, Rodríguez RL. Ordinary least squares regression is indicated for studies of allometry. J Evol Biol. Wiley Online Library; 2017;30:4–12.

14. Prairie YT. Evaluating the predictive power of regression models. Can J Fish Aquat Sci. NRC Research Press; 1996;53:490–2.
